# Supplementary material for: Associations of cardiovascular biomarkers and plasma albumin with exceptional survival to the highest ages
Source: Nat Commun. 2020 Jul 30;11:3820. doi: 10.1038/s41467-020-17636-0 (PMC7393489; doi:10.1038/s41467-020-17636-0)
Supplement: Supplementary file 1 — Supplementary Information [file 41467_2020_17636_MOESM1_ESM.pdf]

Supplementary Information

Associations of Cardiovascular Biomarkers and Plasma Albumin with Exceptional Survival to the Highest Ages

Hirata et al.

Table of Contents

Supplementary Note 1	List of investigators………………………………………………	2
Supplementary Figure 1	Cross-sectional associations of NT-proBNP with age at enrollment by electrocardiographic abnormality………………………………………	4
Supplementary Figure 2	Overall and age-group specific hazard ratios for death from any causes, according to candidate biomarker levels, confined to participants without cardiovascular abnormality…………………………………….	5
Supplementary Figure 3	Overall and age-group specific hazard ratios for death from any causes, according to candidate biomarker levels, confined to participants without highest tertiles of Cystatin C……………………………...…	6
Supplementary Table 1	Characteristics of participants according to age at enrollment…………	7
Supplementary Table 2	Correlation between circulating biomarkers and age by cardiovascular status, confined to female participants………………………………….	10
Supplementary Table 3	Age and sex-adjusted partial correlation coefficients between circulating biomarkers, by cardiovascular status……………………….	11
Supplementary Table 4	Independent factors associated with natural-Log-transformed NT-proBNP in multivariate stepwise linear regression with backward elimination……………………………………………………………	12
Supplementary Table 5	Associations of demographics, traditional cardiovascular risk factors, and plasma albumin levels with all-cause mortality in the entire sample (the Base Model)……………………………………………………..	13
Supplementary Table 6	The least absolute shrinkage and selection operator (LASSO) coefficient profiles of 17 markers associated with mortality in overall and across age groups……………………………………………....................	14
Supplementary Table 7	Independent prognostic markers resulting from the multivariate forward stepwise selection on candidate biomarkers and traditional risk Factors in overall and across age groups………………………………………	15
Supplementary Table 8	Independent prognostic markers resulting from the forced entry models on candidate biomarkers and traditional risk factors in overall and across age groups……………………………………………………………	16
Supplementary Table 9	Age-group specific hazard ratios for death from any cause, according to prognostic biomarkers as categorical variables………………………	17
Supplementary Table 10	Cross-sectional comparison of the levels of prognostic biomarkers and albumin at enrollment among decedent centenarian categories………...	19
Supplementary References……………………………………………………………………		20

Supplementary Note 1: List of Investigators

The Tokyo Centenarian Study and The Japan Semi-supercentenarian Study

Keio University School of Medicine, Tokyo, Japan

Yukiko Abe, B.A.; Yasumichi Arai, M.D., Ph.D. (PI); Yoshinori Ebihara, M.D., Ph.D.; Takumi Hirata, M.D., Ph.D.; Nobuyoshi Hirose, M.D., Ph.D.; Akira Kunitomi, M.D., Ph.D.; Susumu Nakazawa, M.D., PhD.; Hideyuki Okano, M.D., Ph.D.; Takashi Sasaki, Ph.D.; Michiyo Takayama, M.D.; Ph.D.; Toru Takebayashi, M.D., Ph.D.; Ken Yamamura, M.D., Ph.D.; Kimio Yoshimura, M.D., Ph.D.; Shinsuke Yuasa, M.D., Ph.D.

※ Dr. Takumi Hirata moved to Department of Public Health, Hokkaido University Faculty of Medicine, Sapporo, Japan in January 2020.

Tokyo Metropolitan Institute of Gerontology, Tokyo, Japan

Hiroki Inagaki, Ph.D.; Yukie Masui, PhD.

Gifu Pharmaceutical University, Gifu, Japan

Tetsuo Adachi, Ph.D.

Graduate School of Medical Sciences, Kumamoto University, Kumamoto, Japan

Motoyoshi Endo, M.D., Ph.D.; Jun Morinaga, M.D., Ph.D.; Yuichi Oike, M.D., Ph.D.

Graduate School of Human Sciences, Osaka University, Osaka, Japan

Yasuyuki Gondo, PhD.

The Tokyo Oldest Old Survey on Total Health

Keio University School of Medicine, Tokyo, Japan

Yukiko Abe, B.A.; Yasumichi Arai, M.D., Ph.D. (PI); Takumi Hirata, M.D., Ph.D.; Nobuyoshi Hirose, M.D., Ph.D.; Akira Kunitomi, M.D., Ph.D.; Hideyuki Okano, M.D., Ph.D.; Takashi Sasaki, Ph.D.; Michiyo Takayama, M.D.; Ph.D.; Toru Takebayashi, M.D., Ph.D.; Kimio Yoshimura, M.D., Ph.D.; Shinsuke Yuasa, M.D., Ph.D.

Keio University, Kanagawa, Japan

Yuko Oguma, M.D., Ph.D; Midori Takayama, PhD.

Nihon University School of Dentistry, Tokyo, Japan

Yusuke Fukui, DDS., PhD.; Motoko Fukumoto, DDS. PhD.; Nobuhito Gionhaku, DDS, PhD.; Toshimitsu Iinuma, DDS., PhD.(Co-PI); Kazuhiro Komiyama, DDS., PhD.

Gifu Pharmaceutical University, Gifu, Japan
Tetsuo Adachi, Ph.D.

Graduate School of Medical Sciences, Kumamoto University, Kumamoto, Japan
Motoyoshi Endo, M.D., Ph.D.; Jun Morinaga, M.D., Ph.D.; Yuichi Oike, M.D., Ph.D.

Supplementary Figure 1. Cross-sectional associations of NT-proBNP with age at enrollment by electrocardiographic abnormality

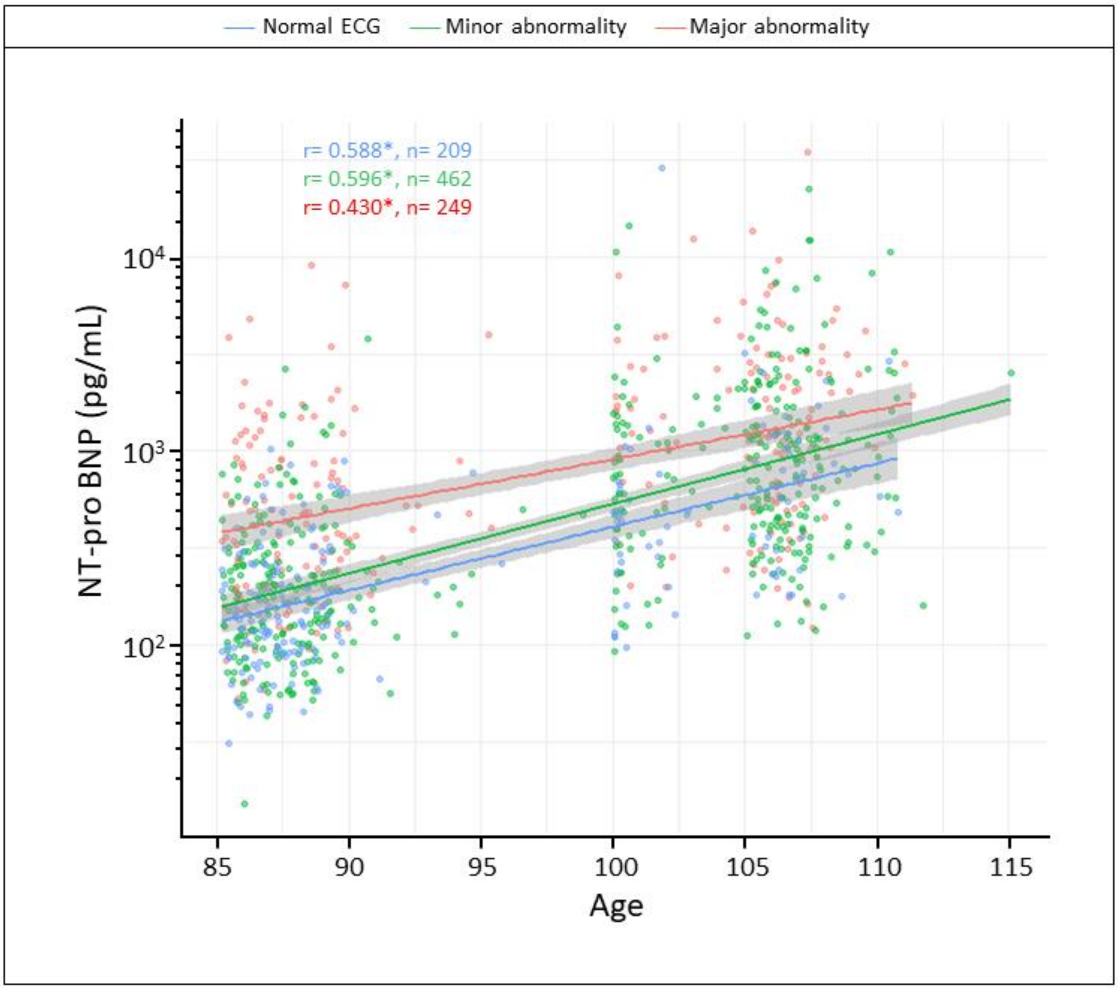

Scatter plots show cross-sectional associations between log-transformed NT-proBNP and age at enrollment according to electrocardiographic (ECG) findings; 1) normal ECG (n=209, blue), 2) minor ECG abnormality (n=462, green), 2) major ECG abnormality (n=249, red). Spearman's correlation coefficients of log-transformed NT-proBNP with age at enrollment were calculated by ECG categories. The solid lines represent correlation lines with 95% confidence interval (shaded area). Major ECG abnormality was defined as old myocardial infarction, pacemaker rhythm, atrial fibrillation or flutter, left ventricular hypertrophy, advanced atrioventricular block, left bundle branch block, and Wolff-Parkinson-White syndrome. Minor ECG abnormality was defined as non-specific ST-T change, first-degree atrioventricular block, left axis deviation, right bundle branch block, non-significant Q wave, poor r progression, sinus bradycardia, sinus tachycardia, premature ventricular contraction, premature atrial contraction, low voltage, and other minor abnormalities (Supplementary Table 1).
* p<0.001.
NT-proBNP, N-terminal pro-brain natriuretic peptide.

Supplementary Figure 2. Overall and age-group specific hazard ratios for death from any causes, according to candidate biomarker levels, confined to participants without cardiovascular abnormality

	Crude			Adjusted		
	HR	95%CI	p	HR	95%CI	p
**Entire cohort**						
NT-proBNP	2.11	(1.91-2.33)	<.001	1.45	(1.23-1.71)	<.001
Erythropoietin	1.06	(0.94-1.19)	.368	0.97	(0.84-1.12)	.712
EC-SOD[a]	1.46	(1.34-1.60)	<.001	1.05	(0.90-1.22)	.550
Adiponectin	1.49	(1.35-1.64)	<.001	1.16	(1.01-1.33)	.030
Interleukin-6[b]	1.82	(1.68-1.98)	<.001	1.35	(1.19-1.53)	<.001
TNF-alpha	1.57	(1.45-1.69)	<.001	1.12	(0.98-1.27)	.108
Angptl2	0.99	(0.90-1.09)	.844	0.91	(0.80-1.04)	.168
Cystatin C	1.52	(1.42-1.62)	<.001	1.35	(1.16-1.59)	<.001
Cholinesterase	0.42	(0.37-0.47)	<.001	0.71	(0.59-0.85)	<.001
**85-99 years**						
NT-proBNP	1.24	(1.01-1.51)	.036	1.27	(1.02-1.58)	.033
Erythropoietin	0.95	(0.76-1.20)	.695	0.97	(0.77-1.23)	.787
EC-SOD[a]	0.95	(0.76-1.18)	.624	0.93	(0.72-1.20)	.575
Adiponectin	1.32	(1.07-1.63)	.011	1.39	(1.09-1.76)	.007
Interleukin-6[b]	1.40	(1.15-1.70)	.001	1.44	(1.16-1.79)	.001
TNF-alpha	1.12	(0.93-1.35)	.242	1.13	(0.92-1.39)	.254
Angptl2	1.15	(0.92-1.42)	.217	1.09	(0.86-1.37)	.471
Cystatin C	1.16	(1.01-1.33)	.040	1.17	(0.93-1.48)	.185
Cholinesterase	0.67	(0.53-0.86)	.001	0.67	(0.51-0.87)	.003
**100-104 years**						
NT-proBNP	1.60	(1.29-1.98)	<.001	1.56	(1.16-2.10)	.003
Erythropoietin	0.98	(0.77-1.25)	.866	1.05	(0.76-1.46)	.778
EC-SOD[a]	1.09	(0.91-1.30)	.334	1.01	(0.76-1.34)	.938
Adiponectin	1.03	(0.86-1.23)	.786	0.80	(0.60-1.06)	.113
Interleukin-6[b]	1.35	(1.14-1.59)	<.001	1.33	(1.04-1.70)	.026
TNF-alpha	1.23	(1.05-1.45)	.012	1.29	(0.95-1.75)	.101
Angptl2	0.93	(0.77-1.12)	.461	0.67	(0.47-0.96)	.027
Cystatin C	1.33	(1.13-1.57)	.001	1.30	(0.88-1.92)	.190
Cholinesterase	0.69	(0.56-0.85)	.001	0.80	(0.57-1.11)	.188
**105 years or older**						
NT-proBNP	1.33	(1.15-1.52)	<.001	1.43	(1.15-1.79)	.002
Erythropoietin	0.97	(0.82-1.16)	.743	0.92	(0.72-1.17)	.494
EC-SOD[a]	0.98	(0.83-1.15)	.803	1.05	(0.84-1.31)	.677
Adiponectin	1.04	(0.91-1.18)	.554	1.06	(0.88-1.27)	.560
Interleukin-6[b]	1.29	(1.14-1.47)	<.001	1.28	(1.07-1.53)	.006
TNF-alpha	1.09	(0.94-1.27)	.230	1.04	(0.85-1.27)	.703
Angptl2	0.90	(0.78-1.04)	.162	0.84	(0.68-1.04)	.112
Cystatin C	1.23	(1.06-1.42)	.005	1.66	(1.27-2.17)	<.001
Cholinesterase	0.74	(0.63-0.87)	<.001	0.83	(0.66-1.05)	.117

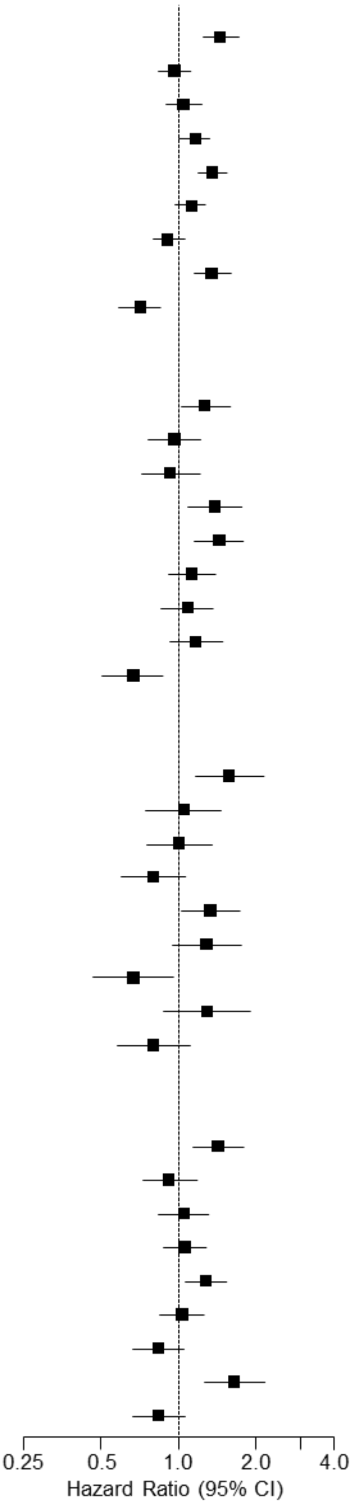

Participants with cardiovascular abnormality at enrollment were excluded from the analysis. Multivariate models were adjusted for sex, age, educational status, current smoking, history of hypertension, hyperlipidemia, diabetes mellitus, chronic kidney disease (stage 3b-5), CRP (≥0.3mg/dL), and low plasma albumin (<3.5g/dL). Each biomarker was entered independently into the models and hazard ratios with 95% confidence intervals and two-sided P values for each biomarker are reported per 1SD increment in natural log-transformed values except cystatin C and cholinesterase. A forest plot shows multivariate-adjusted hazard ratio (squares) and 95% confidence interval (horizontal lines).

[a] Only individuals with 213RR genotype (non-carrier) in *SOD3* (rs1799895) were included in analysis.

[b] CRP (≥0.3mg/dL) was excluded for associations of interleukin-6 with mortality, because it is a downstream biomarker of interleukin-6 pathway.[1]

NT-proBNP, N-terminal pro-brain natriuretic peptide; *EC-SOD,* extracellular superoxide dismutase; *TNF-alpha,* tumor necrosis factor-alpha; *Angptl2,* angiopoietin-like protein 2.

Supplementary Figure 3. Overall and age-group specific hazard ratios for death from any causes, according to candidate biomarker levels, confined to participants without highest tertile of Cystatin C

	Crude			Adjusted		
	HR	95%CI	p	HR	95%CI	p
**Entire cohort**						
NT-proBNP	1.89	(1.72-2.08)	<.001	1.26	(1.10-1.45)	.001
Erythropoietin	1.04	(0.94-1.16)	.407	1.03	(0.92-1.17)	.583
EC-SOD[a]	1.35	(1.23-1.48)	<.001	0.95	(0.84-1.09)	.480
Adiponectin	1.40	(1.28-1.53)	<.001	1.00	(0.89-1.13)	.976
Interleukin-6[b]	1.63	(1.52-1.76)	<.001	1.22	(1.10-1.34)	<.001
TNF-alpha	1.42	(1.33-1.52)	<.001	1.05	(0.95-1.16)	.368
Angptl2	1.01	(0.92-1.10)	.887	0.99	(0.88-1.11)	.838
Cystatin C	1.48	(1.35-1.61)	<.001	1.10	(0.98-1.24)	.115
Cholinesterase	0.42	(0.38-0.47)	<.001	0.73	(0.62-0.85)	<.001
**85-99 years**						
NT-proBNP	1.23	(1.03-1.47)	.024	1.21	(0.98-1.48)	.074
Erythropoietin	0.96	(0.78-1.18)	.680	0.91	(0.73-1.13)	.377
EC-SOD[a]	0.86	(0.70-1.05)	.142	0.96	(0.76-1.22)	.746
Adiponectin	1.14	(0.95-1.37)	.153	1.17	(0.94-1.46)	.159
Interleukin-6[b]	1.27	(1.08-1.49)	.004	1.14	(0.94-1.37)	.190
TNF-alpha	1.05	(0.89-1.25)	.567	1.01	(0.84-1.21)	.946
Angptl2	1.06	(0.88-1.28)	.545	0.92	(0.74-1.13)	.422
Cystatin C	1.22	(1.01-1.47)	.040	1.11	(0.90-1.37)	.337
Cholinesterase	0.69	(0.56-0.86)	.001	0.75	(0.58-0.96)	.025
**100-104 years**						
NT-proBNP	1.35	(1.12-1.64)	.002	1.15	(0.87-1.52)	.310
Erythropoietin	0.97	(0.81-1.16)	.709	1.00	(0.79-1.26)	.976
EC-SOD[a]	1.03	(0.87-1.21)	.743	0.96	(0.74-1.23)	.728
Adiponectin	0.96	(0.82-1.12)	.569	0.80	(0.66-0.97)	.026
Interleukin-6[b]	1.15	(0.99-1.34)	.058	1.13	(0.91-1.40)	.283
TNF-alpha	1.12	(0.98-1.28)	.099	1.13	(0.88-1.46)	.327
Angptl2	1.00	(0.83-1.20)	.986	0.97	(0.74-1.27)	.815
Cystatin C	1.09	(0.92-1.29)	.306	1.06	(0.83-1.37)	.630
Cholinesterase	0.78	(0.65-0.93)	.007	1.08	(0.83-1.42)	.557
**105 years or older**						
NT-proBNP	1.29	(1.14-1.46)	<.001	1.28	(1.08-1.50)	.004
Erythropoietin	1.14	(0.99-1.31)	.063	1.24	(1.05-1.47)	.012
EC-SOD[a]	0.90	(0.78-1.03)	.119	0.94	(0.78-1.13)	.526
Adiponectin	1.03	(0.92-1.15)	.660	1.02	(0.87-1.18)	.835
Interleukin-6[b]	1.27	(1.15-1.42)	<.001	1.24	(1.08-1.43)	.002
TNF-alpha	1.06	(0.94-1.20)	.371	1.02	(0.89-1.18)	.752
Angptl2	1.05	(0.92-1.21)	.467	0.95	(0.80-1.14)	.596
Cystatin C	0.96	(0.86-1.07)	.472	1.09	(0.94-1.28)	.251
Cholinesterase	0.72	(0.62-0.82)	<.001	0.77	(0.64-0.93)	.007

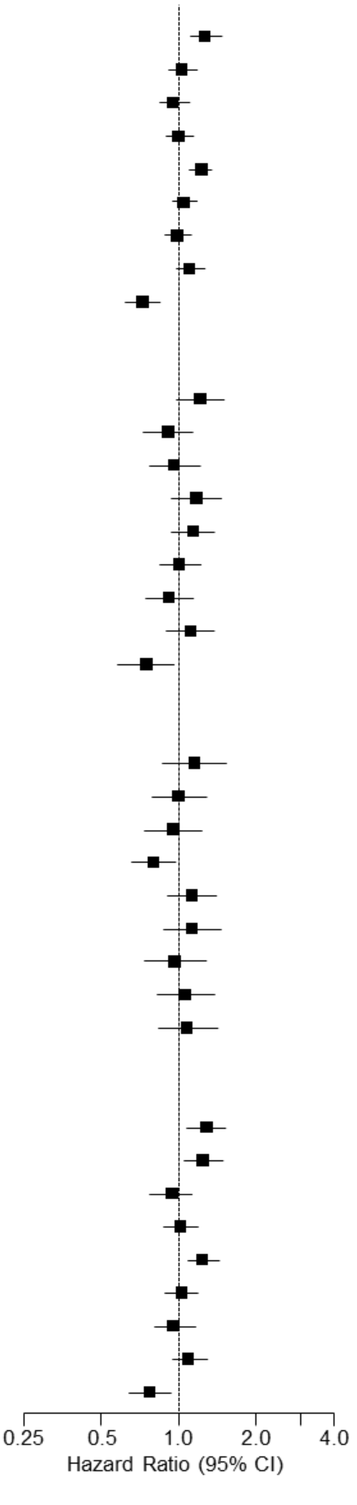

Participants with age-specific highest tertile of cystatin C were excluded from the analysis. Multivariate models were adjusted for sex, age, educational status, current smoking, history of cardiovascular disease, hypertension, hyperlipidemia, diabetes mellitus, chronic kidney disease (stage 3b-5), CRP (≥0.3mg/dL), major ECG abnormality, cardiovascular medications, and low plasma albumin (<3.5g/dL). Each biomarker was entered independently into the models and hazard ratios with 95% confidence intervals and two-sided P values for each biomarker are reported per 1SD increment in natural log-transformed values except cystatin C and cholinesterase. A forest plot shows multivariate-adjusted hazard ratio (squares) and 95% confidence interval (horizontal lines).

[a] Only individuals with 213RR genotype (non-carrier) in *SOD3* (rs1799895) were included in analysis.

[b] CRP (≥0.3mg/dL) was excluded for associations of interleukin-6 with mortality, because it is a downstream biomarker of interleukin-6 pathway.

NT-proBNP, N-terminal pro-brain natriuretic peptide; *EC-SOD,* extracellular superoxide dismutase; *TNF-alpha,* tumor necrosis factor-alpha; *Angptl2,* angiopoietin-like protein 2.

Supplementary Table 1. Characteristics of participants according to age at enrollment

Characteristics	N	Very old (85-99 years)	N	Centenarians (100-104 years)	N	Semi-supercentenarians (105-109 years)	N	Supercentenarians (110+ years)	*P* for trend
Age at enrollment, years (IQR)	531	87.4 (86.3-88.8)	288	100.8 (100.2-102.3)	572	106.6 (105.8-107.4)	36	110.7 (110.4-111.3)	<0.001
Female, no.(%)	531	298 (56.1%)	288	225 (78.1%)	572	502 (87.8%)	36	34 (94.4%)	<0.001
Current smoker, no.(%)	511	36 (7.1%)	282	3 (1.1%)	564	7 (1.2%)	35	1 (2.9%)	<0.001
High education, no.(%)	513	193 (37.6%)	275	61 (22.2%)	540	63 (11.7%)	34	3 (8.8%)	<0.001
Body mass index	528	21.5 ±3.2	187	19.5 ±3.2	353	19.4 ±3.3	21	18.4 ±2.9	<0.001
Barthel index	529	95 ±12	280	48 ±35	564	28 ±28	34	22 ±25	<0.001
Mini-mental state examination	524	26.2 ±4.1	243	13.9 ±8.2	365	7.8 ±7.5	26	5.2 ±6.7	<0.001
**Medical history**									
Coronary heart disease, no.(%)	531	53 (10.0%)	283	41 (14.5%)	566	78 (13.8%)	36	3 (8.3%)	0.124
Stroke, no.(%)	531	92 (17.3%)	283	46 (16.3%)	566	123 (21.7%)	36	2 (5.6%)	0.268
Hypertension, no.(%)	531	334 (62.9%)	287	110 (38.3%)	568	254 (44.7%)	36	14 (38.9%)	<0.001
Hyperlipidemia, no.(%)	530	251 (47.4%)	288	40 (13.9%)	572	83 (14.5%)	36	8 (22.2%)	<0.001
Diabetes Mellitus, no.(%)	531	99 (18.6%)	288	21 (7.3%)	572	32 (5.6%)	36	2 (5.6%)	<0.001
Chronic kidney disease (stage 3b-5), no (%)	530	77 (14.5%)	288	101 (35.1%)	572	214 (37.4%)	36	11 (30.6%)	<0.001
Anemia, no.(%)	531	231 (43.5%)	288	205 (71.2%)	572	387 (67.7%)	36	20 (55.6%)	＜0.001
**Medication**									
Nitrate, no.(%)	527	53 (10.1%)	279	39 (14.0%)	561	79 (14.1%)	32	3 (9.4%)	0.084
Oral anticoagulant, no.(%)	527	20 (3.8%)	279	1 (0.4%)	561	6 (1.1%)	32	0 (0.0%)	<0.001
Antiarrhythmic drug, no.(%)	527	21 (4.0%)	279	3 (1.1%)	561	9 (1.6%)	32	0 (0.0%)	0.007
Digoxin, no.(%)	527	16 (3.0%)	279	11 (3.9%)	561	32 (5.7%)	32	1 (3.1%)	0.050
Diuretics, no.(%)	527	61 (11.6%)	279	62 (22.2%)	561	166 (29.6%)	32	9 (28.1%)	<0.001
Calcium-channel blocker, no.(%)	527	213 (40.4%)	279	47 (16.9%)	561	101 (18.0%)	32	3 (9.4%)	<0.001
ACE inhibitor or ARB, no.(%)	527	157 (29.8%)	279	26 (9.3%)	561	70 (12.5%)	32	6 (18.8%)	<0.001
Beta-blocker, no.(%)	527	47 (8.9%)	279	4 (1.4%)	561	7 (1.3%)	32	0 (0.0%)	<0.001
Antiplatelet, no.(%)	527	141 (26.8%)	279	25 (9.0%)	561	60 (10.7%)	32	1 (3.1%)	<0.001
Statin, (%)	527	81 (15.4%)	279	5 (1.8%)	561	10 (1.8%)	32	1 (3.1%)	<0.001
No Circulatory drugs, no.(%)	527	167 (31.7%)	279	136 (48.8%)	561	262 (46.7%)	32	18 (56.3%)	<0.001
**Clinical presentation**									
Systolic blood pressure (mmHg)	529	143 ±19	269	142 ±23	502	133 ±23	29	131 ±21	<0.001
Diastolic blood pressure (mmHg)	529	77 ±12	262	76 ±13	494	72 ±14	28	71 ±15	<0.001

Continued from Supplementary Table 1

Characteristics	N	Very old (85-99 years)	N	Centenarians (100-104 years)	N	Semi-supercentenarians (105-109 years)	N	Supercentenarians (110+ years)	*P* for trend
Pulse (/min)	523	75 ±10	225	74 ±10	455	75 ±13	24	79 ±14	0.638
Heart murmurs, no.(%)	528	108 (20.5%)	274	121 (44.2%)	553	201 (36.4%)	30	7 (23.3%)	<0.001
Wheeze, no.(%)	529	11 (2.1%)	274	10 (3.7%)	552	11 (2.0%)	30	2 (6.7%)	0.714
Ankle edema, no.(%)	530	90 (17.0%)	274	49 (17.9%)	552	124 (22.5%)	30	10 (33.3%)	0.006
**Electrocardiogram**									
Pulse on Electrocardiogram /min	521	70 ±11	193	73 ±12	453	73 ±15	29	75 ±17	0.001
Normal, (%)	521	151 (29.0%)	193	41 (21.2%)	453	57 (12.6%)	29	4 (13.8%)	<0.001
Major abnormality, (%)	521	134 (25.7%)	193	50 (25.9%)	453	139 (30.7%)	29	7 (24.1%)	0.140
Old myocardial infarction, (%)	521	21 (4.0%)	193	8 (4.2%)	453	52 (11.5%)	29	4 (13.8%)	<0.001
Pacemaker rhythm, (%)	521	6 (1.2%)	193	3 (1.6%)	453	5 (1.1%)	29	2 (6.9%)	0.409
Atrial fibrillation, (%)	521	23 (4.4%)	193	13 (6.7%)	453	29 (6.4%)	29	1 (3.5%)	0.257
Atrial flutter, (%)	521	2 (0.4%)	193	1 (0.5%)	453	2 (0.4%)	29	0 (0.0%)	0.984
Left ventricular hypertrophy, (%)	521	90 (17.3%)	193	21 (10.9%)	453	56 (12.4%)	29	1 (3.5%)	0.008
Advanced atrioventricular block, (%)	521	0 (0.0%)	193	2 (1.0%)	453	5 (1.1%)	29	0 (0.0%)	0.045
Left bundle branch block, (%)	521	5 (1.0%)	193	8 (4.2%)	453	9 (2.0%)	29	0 (0.0%)	0.346
WPW syndrome, (%)	521	1 (0.2%)	193	0 (0.0%)	453	2 (0.4%)	29	0 (0.0%)	0.535
Minor abnormality, (%)	521	236 (45.3%)	193	102 (52.9%)	453	257 (56.7%)	29	18 (62.1%)	<0.001
Non-specific ST-T change, (%)	521	87 (16.7%)	193	53 (27.5%)	453	126 (27.8%)	29	8 (27.6%)	<0.001
First-degree atrioventricular block, (%)	521	74 (14.2%)	193	20 (10.4%)	453	99 (21.9%)	29	9 (31.0%)	<0.001
Left anterior hemiblock, (%)	521	30 (5.8%)	193	7 (3.6%)	453	44 (9.7%)	29	6 (20.7%)	0.002
Right bundle branch block, (%)	521	83 (15.9%)	193	33 (17.1%)	453	97 (21.4%)	29	5 (17.2%)	0.043
Left axis deviation, (%)	521	26 (5.0%)	193	6 (3.1%)	453	35 (7.7%)	29	5 (17.2%)	0.013
Sinus bradycardia, (%)	521	11 (2.1%)	193	5 (2.6%)	453	14 (3.1%)	29	0 (0.0%)	0.529
Sinus tachycardia, (%)	521	5 (1.0%)	193	4 (2.1%)	453	9 (2.0%)	29	1 (3.5%)	0.136
Low voltage in limb lead, (%)	521	4 (0.8%)	193	1 (0.5%)	453	7 (1.6%)	29	2 (6.9%)	0.045
Poor r progression, (%)	521	1 (0.2%)	193	1 (0.5%)	453	16 (3.5%)	29	4 (13.8%)	<0.001
Non-significant Q wave, (%)	521	1 (0.2%)	193	4 (2.1%)	453	12 (2.7%)	29	0 (0.0%)	0.004
Premature atrial contractions, (%)	521	41 (7.9%)	193	20 (10.4%)	453	73 (16.1%)	29	3 (10.3%)	<0.001
Premature ventricular contractions, (%)	521	16 (3.1%)	193	7 (3.6%)	453	20 (4.4%)	29	1 (3.5%)	0.306
Other minor abnormalities [a], (%)	521	10 (1.9%)	193	3 (1.6%)	453	4 (0.9%)	29	0 (0.0%)	0.134

Continued from Supplementary Table 1

Characteristics	N	Very old (85-99 years)	N	Centenarians (100-104 years)	N	Semi-supercentenarians (105-109 years)	N	Supercentenarians (110+ years)	*P* for trend
**Cardioprotective factors**									
NT-proBNP, ng/L (IQR)	475	195 (115-392)	199	687 (376-1360)	385	960 (465-1900)	21	1530 (587-2540)	<0.001
Erythropoietin, mIU/mL (IQR)	415	10.3 (7.8-14.2)	199	10.4 (7.6-14.4)	385	11.3 (8.2-16.3)	21	12.2 (8.4-15.2)	0.031
SOD3 R213G genotype (rs1799895)									
non-carrier (RR)	530	475 (89.6%)	288	265 (92.0%)	565	518 (91.7%)	35	33 (94.3%)	0.661[c]
heterozygotes (RG)	530	51 (9.6%)	288	20 (6.9%)	565	45 (8.0%)	35	2 (5.7%)	
homozygotes (GG)	530	4 (0.8%)	288	3 (1.0%)	565	2 (0.4%)	35	0 (0.0%)	
EC-SOD concentration[b], ng/mL (IQR)	448	106 (88-127)	222	137 (113-169)	324	146 (121-180)	20	168 (124-203)	<0.001
Adiponectin, ng/mL (IQR)	529	12.0 (7.3-19.2)	271	16.9 (12.5-23.2)	537	18.5 (13.3-25.0)	34	20.2 (16.4-23.9)	<0.001
**Inflammatory mediators**									
Interleukin-6, pg/mL (IQR)	529	1.7 (1.3-2.5)	272	2.9 (2.3-4.3)	545	3.4 (2.3-5.4)	34	4.9 (3.0-7.3)	<0.001
TNF-alpha, pg/mL (IQR)	529	2.2 (1.9-2.8)	272	3.4 (2.7-4.2)	536	4.2 (3.0-5.6)	32	3.9 (2.5-4.9)	<0.001
Angptl2, ng/mL (IQR)	529	4.1 (3.2-5.3)	252	3.9 (3.2-5.0)	409	4.2 (3.4-5.1)	25	4.1 (3.5-5.1)	0.461
**Organ reserve**									
Cystatin C (mg/dL)	524	1.26 ±0.51	265	1.63 ±0.52	522	1.80 ±0.54	31	1.84 ±0.60	<0.001
Cholinesterase (IU/L)	531	277 ±68	287	214 ±56	569	196 ±58	36	177 ±42	<0.001
**Traditional Risk Factors (Continuous Variables)**									
HDL cholesterol (mg/dL)	530	58.8 ±14.7	288	52.5 ±13.3	572	45.8 ±11.9	36	45.9 ±11.8	<0.001
LDL cholesterol (mg/dL)	525	117. ±26.9	288	99.8 ±28.2	572	102.0 ±28.1	36	96.2 ±29.3	<0.001
Hemoglobin A1c (%)	528	5.94 ±0.76	273	5.70 ±0.70	560	5.52 ±0.51	36	5.51 ±0.36	<0.001
Creatinine (mg/dL)	530	0.84 ±0.51	288	0.90 ±0.45	572	0.87 ±0.43	36	0.82 ±0.34	0.684
eGFRcr (mL/min/1.73m^2)	530	61.9 ±16.4	288	55.3 ±22.6	572	55.9 ±26.0	36	55.2 ±22.0	<0.001
CRP, mg/dL (IQR)	531	0.09 (0.04-0.19)	287	0.16 (0.05-0.46)	572	0.25 (0.09-0.66)	36	0.34 (0.13-0.88)	<0.001
Albumin (g/dL)	531	4.1 ±0.3	287	3.6 ±0.4	572	3.4 ±0.4	36	3.2 ±0.4	<0.001

IQR, inter-quartile range; *ACE*, angiotensin-converting enzyme; *ARB*, Angiotensin II Receptor Blocker; *WPW*, Wolf-Parkinson-White; *NT-proBNP*, N-terminal pro-brain natriuretic peptide; *EC-SOD,* extracellular superoxide dismutase; *TNF-alpha*, tumor necrosis factor-alpha; *Angptl2*, angiopoietin-like protein 2; *eGFRcr,* estimated glomerular filtration rate based on plasma creatinine; HDL, high-density lipoprotein; *LDL*, low-density lipoprotein; CRP, C-reactive protein.

Plus-minus values are means ± SD. Trends in each characteristic of participants across four age groups were analyzed using the trend test for continuous variables, and the Cochran-Armitage test for trend for categorical variables.

[a] Other minor abnormalities include counter-clockwise rotation, interventricular conduction delay, ectopic atrial rhythm, and right atrial overload.

[b] Only individuals with RR genotype in *SOD3* (rs1799895) were included in analysis.

[c] P value for Pearson's Chi-squared test.

Supplementary Table 2. Correlation between circulating biomarkers and age by cardiovascular status, confined to female participants

	No Cardiovascular abnormality			Cardiovascular abnormality		
	Spearman's correlation	p	n	Spearman's correlation	p	n
NT-proBNP	.740	.000	510	.488	.000	371
Erythropoietin	.237	.000	464	.069	.195	357
EC-SOD[a]	.529	.000	498	.384	.000	338
Adiponectin	.357	.000	637	.219	.000	477
Interleukin-6	.638	.000	642	.393	.000	482
TNF-alpha	.587	.000	635	.469	.000	478
Angptl2	.072	.088	563	.081	.108	399
Cystatin C	.692	.000	608	.473	.000	468
Cholinesterase	-.659	.000	664	-.503	.000	499
LDL-cholesterol	-.330	.000	663	-.219	.000	499
Creatinine	.216	.000	664	.088	.050	501
Albumin	-.733	.000	665	-.570	.000	500

NT-proBNP, N-terminal pro-brain natriuretic peptide; *EC-SOD,* extracellular superoxide dismutase; *TNF-alpha,* tumor necrosis factor-alpha; *Angptl2,* angiopoietin-like protein 2; *LDL,* low-density lipoprotein.

Analytic cohort was confined to women only. All the biomarkers were assessed at the time of enrollment. Spearman's correlation coefficients and two-sided P values between biomarkers and age at enrollment were calculated according to cardiovascular status. Unrelated family members of the centenarians (spouses of the first-degree offspring of the centenarians) aged between 48 and 85 years (mean age, 72.2 years) were included as younger control group (n=126 at the maximum). Characteristics of this population are described in Supplementary reference 2. Population sizes for the twelve biomarkers differ due to variation in the bio-banking of samples. Participants were considered to have a cardiovascular abnormality when one or more of the following criteria were fulfilled: 1) a history of coronary heart disease or stroke, 2) cardiovascular medication use (i.e., nitrate, oral anticoagulant, antiarrhythmic drug, or digoxin), and 3) a major electrocardiographic abnormality (Table 1). Classification of cardiovascular abnormality in unrelated family of centenarians was based on medical history and medication list because of lack of ECG assessment in this population.

[a] Only individuals with 213RR genotype (non-carrier) in *SOD3* (rs1799895) were included in the analysis.

Supplementary Table 3. Age and sex-adjusted partial correlation coefficients between circulating biomarkers, by cardiovascular status

	NT-proBNP	Erythropoietin	EC- SOD	Adiponectin	Interleukin-6	TNF-alpha	Angptl2	Cystatin C	Cholinesterase	CRP
**Whole sample**										
Erythropoietin	.168 (.000)									
EC- SOD[a]	.237 (.000)	.080 (.029)								
Adiponectin	.183 (.000)	.050 (.118)	.345 (.000)							
Interleukin-6	.204 (.000)	.144 (.000)	-.090 (.004)	-.018 (.516)						
TNF-alpha	.196 (.000)	.028 (.376)	-.004 (.910)	.034 (.216)	.222 (.000)					
Angptl2	.035 (.247)	.057 (.067)	-.012 (.732)	-.106 (.000)	.132 (.000)	.059 (.042)				
Cystatin C	.394 (.000)	.017 (.594)	.249 (.000)	.037 (.184)	.143 (.000)	.233 (.000)	.051 (.081)			
Cholinesterase	-.193 (.000)	-.124 (.000)	.015 (.644)	-.152 (.000)	-.199 (.000)	-.094 (.000)	.085 (.003)	-.019 (.488)		
CRP	.191 (.000)	.118 (.000)	-.185 (.000)	-.101 (.000)	.541 (.000)	.131 (.000)	.181 (.000)	.103 (.000)	-.158 (.000)	
Albumin	-.145 (.000)	-.149 (.000)	.153 (.000)	-.023 (.398)	-.361 (.000)	-.196 (.000)	-.094 (.001)	-.045 (.100)	.409 (.000)	-.402 (.000)
**No Cardiovascular abnormality**										
Erythropoietin	.151 (.000)									
EC- SOD[a]	.255 (.000)	.100 (.044)								
Adiponectin	.140 (.001)	.048 (.283)	.317 (.000)							
Interleukin-6	.208 (.000)	.121 (.006)	-.141 (.001)	-.035 (.354)						
TNF-alpha	.198 (.000)	.088 (.047)	-.018 (.676)	.041 (.285)	.240 (.000)					
Angptl2	.042 (.326)	.084 (.056)	.026 (.575)	-.088 (.029)	.141 (.000)	.149 (.000)				
Cystatin C	.450 (.000)	.059 (.178)	.258 (.000)	.028 (.462)	.163 (.000)	.255 (.000)	.055 (.178)			
Cholinesterase	-.168 (.000)	-.089 (.042)	.037 (.395)	-.120 (.002)	-.181 (.000)	-.114 (.003)	.070 (.080)	-.046 (.232)		
CRP	.216 (.000)	.099 (.024)	-.223 (.000)	-.155 (.000)	.565 (.000)	.182 (.000)	.170 (.000)	.116 (.002)	-.128 (.000)	
Albumin	-.145 (.000)	-.133 (.002)	.184 (.000)	.004 (.924)	-.361 (.000)	-.238 (.000)	-.081 (.042)	-.067 (.078)	.432 (.000)	-.358 (.000)

NT-proBNP, N-terminal pro-brain natriuretic peptide; *EC-SOD,* extracellular superoxide dismutase; *CRP,* C-reactive protein; *TNF-alpha,* tumor necrosis factor-alpha; *Angptl2,* angiopoietin-like protein 2.
Values displayed are partial correlation coefficients (two-sided P values) after adjustment of age and sex. Logarithmic transformation was performed for NT-proBNP, erythropoietin, EC-SOD, Adiponectin, interleukin-6, TNF-alpha, and Angptl2, and CRP.
[a] Only individuals with 213RR genotype (non-carrier) in *SOD3* (rs1799895) were included in analysis.

Supplementary Table 4. Independent factors associated with natural-log-transformed NT-proBNP in multivariate stepwise linear regression with backward elimination

	Univariate β coefficient	P	Multivariate β coefficient[a]	p
Age	.079	.000	.033	.000
Sex (female)	.460	.000	.106	.170
Smoking	-.440	.026	.259	.088
Cardiovascular disease	.263	.001	.147	.069
Diabetes	-.390	.001	…	…
Hypertension	-.147	.046	…	…
Hyperlipidemia	-.587	.000	…	…
Anemia	.605	.000	…	…
Major ECG abnormality	.755	.000	.597	.000
Old myocardial infarction[b]	.503	.002	.369	.022
Atrial fibrillation [b]	1.16	.000	.817	.000
Cardiovascular Medication	.531	.000	.192	.044
Nitrate[c]	.446	.000	…	…
Digitalis[c]	.656	.000	.271	.123
Diuretic[c]	.644	.000	…	…
Cystatin C	.669	.000	.351	.000
Erythropoietin	.214	.000	.133	.000
EC-SOD	.529	.000	.172	.000
Interleukin-6	.496	.000	.121	.004
Cholinesterase	-.519	.000	-.111	.003
Adiponectin	.414	.000	.089	.018
TNF-alpha	.470	.000	.071	.095
Albumin	-.572	.000	…	…

NT-proBNP, N-terminal pro-brain natriuretic peptide; *ECG,* electrocardiogram; *EC-SOD,* extracellular superoxide dismutase; *TNF-alpha,* tumor necrosis factor-alpha.

Multivariate stepwise linear regression with backward elimination analysis was performed to determine the independent correlates of ln NT-proBNP levels. Variables with two-sided $p < 0.05$ in univariate analysis were incorporated into the multivariate linear regression model. R^2=0.580, adjusted R^2=0.572.

[a] β coefficients for each biomarker are reported per 1SD increment in natural log-transformed values except cystatin C, cholinesterase, and albumin.

[b] β coefficients were calculated when major ECG abnormality was substituted by old myocardial infarction or atrial fibrillation in the same model.

[c] β coefficients were calculated when cardiovascular medication was substituted by nitrates, digitalis, or diuretic in the same model

Supplementary Table 5. Associations of demographics, traditional cardiovascular risk factors, and plasma albumin levels with all-cause mortality in the entire sample (the Base Model)

	Univariate			Model 1[a]			Model 2[b]			Model 3[c]		
	HR	(95%CI)	p	HR	(95%CI)	p	HR	(95%CI)	p	HR	(95%CI)	p
Age (per year)	1.12	(1.11-1.13)	.000	1.13	(1.12-1.14)	.000	1.12	(1.10-1.13)	.000	1.11	(1.09-1.12)	.000
Sex	1.56	(1.34-1.81)	.000	0.80	(0.67-0.94)	.007	0.89	(0.74-1.06)	.192	0.85	(0.71-1.02)	.081
Education	0.53	(0.45-0.62)	.000	0.91	(0.76-1.08)	.276	0.86	(0.71-1.06)	.154	0.89	(0.72-1.08)	.241
Current Smoking	0.61	(0.41-0.89)	.011	1.36	(0.91-2.03)	.132	1.32	(0.87-1.98)	.188	1.25	(0.83-1.88)	.287
Cardiovascular disease	1.29	(1.12-1.47)	.000				1.17	(0.98-1.39)	.083	1.16	(0.98-1.38)	.083
Diabetes	0.71	(0.58-0.88)	.002				1.37	(1.08-1.75)	.010	1.35	(1.06-1.72)	.015
Hypertension	0.68	(0.60-0.77)	.000				0.85	(0.72-0.99)	.033	0.88	(0.75-1.03)	.103
Hyperlipidemia	0.43	(0.37-0.50)	.000				0.84	(0.69-1.02)	.076	0.87	(0.71-1.06)	.171
Chronic kidney disease (stage 3b-5)	1.60	(1.40-1.83)	.000				1.02	(0.86-1.21)	.801	1.08	(0.91-1.28)	.379
CRP (≥0.3mg/dL)	2.44	(2.14-2.77)	.000				1.45	(1.24-1.69)	.000	1.34	(1.14-1.58)	.000
Major ECG abnormality	1.18	(1.01-1.38)	.034				1.16	(0.99-1.37)	.074	1.17	(0.99-1.38)	.056
Cardiovascular medication	1.22	(1.04-1.43)	.016				1.18	(0.96-1.45)	.113	1.18	(0.96-1.45)	.112
Albumin (<3.5g/L)	3.99	(3.49-4.57)	.000							1.51	(1.25-1.81)	.000

CRP, C-Reactive protein; *ECG,* electrocardiogram.

Hazard ratios (HR) and 95% confidence intervals (CI), and two-sided P values were calculated with the use of multivariate Cox proportional hazard models.

[a] Model 1: adjusted for age, sex, educational status, and current smoking.

[b] Model 2: adjusted for the covariates in Model 1 plus history of cardiovascular disease, hypertension, hyperlipidemia, diabetes mellitus, chronic kidney disease (stage 3b-5), elevated CRP (>0.3mg/dL), major ECG abnormality, and cardiac medication (nitrates, antiarrhythmic drugs, warfarin, and digitalis).

[c] Model 3: adjusted for the covariates in Model 2 plus low plasma albumin levels (<3.5g/dL).

Supplementary Table 6.The least absolute shrinkage and selection operator (LASSO) coefficient profiles of 17 markers associated with mortality in overall and across age groups

	a. Entire cohort (n=836)	b. 85-99 years (n=438)	c. 100-104 years (n=124)	d. 105 years or older (n=274)
	Hazard ratio	Hazard ratio	Hazard ratio	Hazard ratio
NT-proBNP	1.22	1.02	1.12	1.20
Interleukin-6	1.04	1.11	…	…
Cystatin C	1.03	1.06	1.01	…
Cholinesterase	0.86	0.84	…	0.95
Creatinine	…	…	…	…
CRP	1.05	1.05	…	1.04
Albumin	0.74	0.79	0.89	0.80
Age	1.08	1.06	…	1.01
Sex (female)	0.82	…	0.97	…
High education	0.86	…	…	…
Current Smoking	1.36	…	…	1.57
Cardiovascular disease	1.18	1.10	…	…
Diabetes	1.25	…	…	1.03
Hypertension	0.86	…	0.82	…
Hyperlipidemia	…	0.92	…	…
Major ECG abnormality	1.07	…	…	1.14
Cardiac medications	…	…	…	…

NT-proBNP, N-terminal pro-brain natriuretic peptide; *CRP,* C-reactive protein; *ECG,* electrocardiogram.
To identify the best overall set of prognostic markers, the prognostic biomarkers identified in Fig 4 (NT-proBNP, interleukin-6, cystatin C, and cholinesterase) and Fig 5 (creatinine, CRP, and albumin) were combined with clinical covariates in the base model; sex, age, educational status, current smoking, history of cardiovascular disease, hypertension, hyperlipidemia, diabetes mellitus, major ECG abnormality, and cardiovascular medications. To standardize the number of participants for the multiple biomarker-risk factor comparisons, we restricted analyses to participants with complete data on all biomarkers. LASSO shrinks coefficients for weaker predictors toward zero (denoted as …). The degree of shrinkage is determined by an optimal parameter lambda.mins, the value of lambda that gives minimum mean cross-validated error are 0.00947, 0.03622, 0.12483, and 0.10931 for the entire cohort (a), those aged 85-99 years at enrollment (b), 100-104 years (c), and 105 years or older (d). LASSO coefficients are converted to hazard ratios as convenient for comparison with the results from the stepwise analysis (Supplementary Table 7).

Supplementary Table 7. Independent prognostic markers resulting from the multivariate forward stepwise selection on candidate biomarkers and traditional risk factors in overall and across age groups

	Final Model (Stepwise Entry)		
	HR	95%CI	P value
**a. Entire cohort**			
Age	1.09	(1.07-1.10)	<.001
Albumin	0.73	(0.64-0.84)	<.001
NT-proBNP	1.25	(1.13-1.40)	<.001
Sex (female)	0.77	(0.62-0.95)	.017
Cholinesterase	0.84	(0.74-0.96)	.010
Diabetes	1.36	(1.02-1.81)	.038
CVD	1.24	(1.02-1.50)	.031
Hypertension	0.84	(0.70-1.01)	.057
Smoking	1.51	(0.95-2.40)	.083
Education	0.81	(0.64-1.02)	.074
CRP	1.07	(0.97-1.19)	.166
**b. 85-99 years**			
Albumin	0.74	(0.61-0.88)	.001
Cholinesterase	0.74	(0.60-0.90)	.003
Interleukin-6	1.22	(1.04-1.42)	.013
Age	1.10	(1.02-1.19)	.013
Cystatin-C	1.20	(1.01-1.44)	.043
CVD	1.37	(0.96-1.96)	.087
Smoking	1.57	(0.88-2.78)	.124
**c. 100-104 years**			
NT-proBNP	1.35	(1.11-1.64)	.003
HBP	0.60	(0.40-0.89)	.010
Albumin	0.78	(0.65-0.94)	.009
Sex (female)	0.70	(0.46-1.08)	.104
**d. 105 years or older**			
NT-proBNP	1.27	(1.12-1.43)	<.001
Albumin	0.71	(0.61-0.82)	<.001
Current smoking	2.94	(1.19-7.26)	.020
Major ECG abnormality	1.35	(1.02-1.80)	.038

NT-proBNP, N-terminal pro-brain natriuretic peptide; *CRP,* C-reactive protein; *ECG,* electrocardiogram.

Hazard ratios (HR) and 95% confidence intervals (CI), and two-sided P values were calculated with the use of multivariate Cox proportional hazard models, where all of prognostic biomarkers identified in Fig 4 (NT-proBNP, interleukin-6, cystatin C, and cholinesterase) and in Fig 5 (creatinine, CRP, and albumin) were combined with clinical covariates in the base model; sex, age, educational status, current smoking, history of cardiovascular disease, hypertension, hyperlipidemia, diabetes mellitus, major ECG abnormality, and cardiovascular medications. (the final model).Significant prognostic markers are identified by using multivariate forward stepwise selection ($p<0.20$) for whole sample combined (a), those aged 85-99 years at enrollment (b), 100-104 years (c), and 105 years or older (d).

Supplementary Table 8. Independent prognostic markers resulting from the forced entry models on candidate biomarkers and traditional risk factors in overall and across age groups (the Final Model)

	a. Entire cohort			b. 85-99 years			c. 100-104 years			d. 105 years or older		
	HR	95%CI	p	HR	95%CI	p	HR	95%CI	p	HR	95%CI	p
NT-proBNP	1.21	(1.07-1.37)	.002	1.10	(0.90-1.35)	.359	1.28	(0.95-1.72)	.108	1.23	(1.07-1.41)	.003
Interleukin-6	1.03	(0.93-1.15)	.552	1.11	(0.92-1.34)	.278	1.05	(0.83-1.33)	.657	1.03	(0.88-1.20)	.719
Cystatin C	1.03	(0.85-1.25)	.760	1.72	(1.07-2.76)	.024	0.96	(0.65-1.42)	.834	0.92	(0.71-1.19)	.524
Cholinesterase	0.85	(0.74-0.97)	.016	0.78	(0.63-0.96)	.020	1.06	(0.85-1.33)	.617	0.90	(0.76-1.08)	.266
Creatinine	1.01	(0.84-1.20)	.950	0.67	(0.42-1.07)	.094	1.31	(0.89-1.91)	.169	1.01	(0.75-1.34)	.971
CRP	1.06	(0.95-1.19)	.273	1.10	(0.91-1.31)	.323	0.96	(0.75-1.23)	.753	1.04	(0.88-1.23)	.622
Albumin	0.73	(0.63-0.85)	<.001	0.77	(0.64-0.93)	.007	0.70	(0.54-0.91)	.009	0.75	(0.62-0.91)	.004
Age	1.09	(1.07-1.11)	<.001	1.09	(1.00-1.18)	.045	1.04	(0.89-1.22)	.632	1.05	(0.96-1.15)	.265
Sex (female)	0.78	(0.62-0.99)	.041	0.80	(0.54-1.20)	.283	0.82	(0.50-1.34)	.426	0.89	(0.58-1.35)	.573
High education	0.82	(0.65-1.04)	.100	0.77	(0.53-1.12)	.167	1.02	(0.62-1.68)	.939	0.91	(0.59-1.40)	.656
Current Smoking	1.52	(0.95-2.43)	.080	1.40	(0.76-2.56)	.282	1.05	(0.23-4.78)	.946	2.75	(1.08-7.03)	.034
Cardiovascular disease	1.25	(1.01-1.54)	.042	1.63	(1.07-2.49)	.024	1.72	(1.02-2.91)	.042	1.00	(0.73-1.37)	.999
Diabetes	1.35	(1.01-1.81)	.044	1.26	(0.84-1.90)	.265	0.56	(0.23-1.33)	.188	1.48	(0.85-2.57)	.162
Hypertension	0.84	(0.70-1.01)	.070	0.84	(0.58-1.23)	.370	0.55	(0.35-0.87)	.010	0.92	(0.70-1.21)	.548
Hyperlipidemia	0.98	(0.78-1.24)	.871	0.76	(0.54-1.08)	.128	1.82	(0.92-3.58)	.085	1.19	(0.81-1.76)	.367
Major ECG abnormality	1.10	(0.89-1.36)	.358	0.79	(0.53-1.19)	.265	0.92	(0.55-1.55)	.752	1.31	(0.97-1.77)	.081
Atrial fibrillation[a]	1.15	(0.80-1.66)	.438	1.13	(0.48-2.65)	.779	0.77	(0.36-1.66)	.504	1.75	(1.03-2.98)	.038
OMI[a]	1.27	(0.90-1.79)	.171	1.04	(0.41-2.62)	.930	0.28	(0.07-1.10)	.068	1.63	(1.09-2.43)	.017
Cardiac medications	0.96	(0.75-1.22)	.717	0.90	(0.55-1.49)	.694	0.67	(0.37-1.24)	.205	1.18	(0.83-1.68)	.349

NT-proBNP, N-terminal pro-brain natriuretic peptide; *CRP,* C-reacvtive protein; *ECG,* electrocardiogram; *OMI,* old myocardial infarction.
Hazard ratios (HR) and 95% confidence intervals (CI), and two-sided P values were calculated with the use of multivariate Cox proportional hazard models, where all of prognostic biomarkers significantly associated with mortality in Fig 4 and Fig 5, and clinical covariates in the base model were entered (the final model). Significant prognostic markers are identified by using forced entry models for whole sample combined (a), those aged 85-99 years at enrollment (b) , 100-104 years (c), and 105 years or older (d). Hazard ratios for each biomarker are reported per 1SD increment in natural log-transformed values except cystatin C, cholinesterase, creatinine, and albumin.
[a] Atrial fibrillation or OMI was entered into the final model, instead of Major ECG abnormality.

Supplementary Table 9. Age-group specific hazard ratios for death from any cause, according to prognostic biomarkers as categorical variables

		Crude			Adjusted		
	Event/No at	HR	95%CI	p	HR	95%CI	p
**a 85-99 years at enrollment**							
NT-proBNP, tertiles[a]							
<136 pg/mL	42/160	1.00	Reference		1.00	Reference	
136-296 pg/mL	58/157	1.56	(1.05-2.32)	.028	1.53	(1.00-2.34)	.049
>296 pg/mL	66/158	1.85	(1.25-2.72)	.002	1.94	(1.24-3.04)	.004
NT-proBNP, dichotomize[b]							
<1800pg/mL	160/464	1.00	Reference		1.00	Reference	
≥1800pg/mL	6/11	2.54	(1.12-5.74)	.025	1.57	(0.59-4.17)	.370
Interleukin-6, tertiles[a,c]							
<1.41 pg/mL	51/177	1.00	Reference		1.00	Reference	
1.41-2.16 pg/mL	68/177	1.44	(0.99-2.07)	.050	1.32	(0.89-1.98)	.171
>2.16 pg/mL	71/175	1.59	(1.11-2.28)	.011	1.24	(0.83-1.86)	.288
Cystatin C, tertiles[a]							
<1.08 mg/dL	52/177	1.00	Reference		1.00	Reference	
1.08-1.27 mg/dL	64/173	1.37	(0.95-1.98)	.089	1.22	(0.81-1.83)	.336
>1.27 mg/dL	71/174	1.62	(1.13-2.31)	.009	1.44	(0.92-2.27)	.114
Cholinesterase, tertiles[a]							
<249 U/L	87/180	1.00	Reference		1.00	Reference	
249-295 U/L	57/176	0.57	(0.41-0.80)	.001	0.61	(0.42-0.89)	.011
>295 U/L	47/175	0.46	(0.32-0.65)	<.001	0.54	(0.36-0.82)	.003
**b 100-104 years at enrollment**							
NT-proBNP, tertiles[a]							
<454 pg/mL	60/67	1.00	Reference		1.00	Reference	
454-1050 pg/mL	63/66	1.27	(0.89-1.82)	.192	0.80	(0.47-1.36)	.410
>1050 pg/mL	63/66	2.46	(1.69-3.57)	<.001	1.83	(1.03-3.26)	.041
NT-proBNP, dichotomize[b]							
<1800pg/mL	154/165	1.00	Reference		1.00	Reference	
≥1800pg/mL	32/34	2.02	(1.37-2.99)	<.001	1.71	(0.94-3.10)	.079
Interleukin-6, tertiles[a,c]							
<2.46 pg/mL	88/92	1.00	Reference		1.00	Reference	
2.46-3.75 pg/mL	87/90	1.13	(0.84-1.52)	.415	0.94	(0.62-1.41)	.756
>3.75 pg/mL	86/90	1.44	(1.07-1.94)	.017	1.09	(0.74-1.61)	.674
Cystatin C, tertiles[a]							
<1.34 mg/dL	87/90	1.00	Reference		1.00	Reference	
1.34-1.72 mg/dL	82/87	1.19	(0.87-1.62)	.276	1.13	(0.73-1.76)	.585
>1.72 mg/dL	83/88	1.76	(1.29-2.42)	<.001	1.42	(0.77-2.61)	.261
Cholinesterase, tertiles[a]							
<190 U/L	91/96	1.00	Reference		1.00	Reference	
190-234 U/L	90/96	0.52	(0.39-0.70)	<.001	0.71	(0.46-1.08)	.113
>234 U/L	89/95	0.42	(0.31-0.57)	<.001	0.75	(0.47-1.18)	.212
**c 105 years or older at enrollment**							
NT-proBNP, tertiles[a]							
<585 pg/mL	104/130	1.00	Reference		1.00	Reference	
585-1340 pg/mL	118/133	1.74	(1.33-2.27)	<.001	1.33	(0.95-1.85)	.095
>1340 pg/mL	125/131	2.08	(1.59-2.70)	<.001	1.91	(1.36-2.68)	<.001

Continued from Supplementary Table 9

		Crude			Adjusted		
	Event/No at	HR	95%CI	p	HR	95%CI	p
NT-proBNP, dichotomize[b]							
<1800pg/mL	241/284	1.00	Reference		1.00	Reference	
≥1800pg/mL	106/110	1.57	(1.24-1.98)	<.001	1.68	(1.25-2.27)	.001
Interleukin-6, tertiles[a,c]							
<2.64 pg/mL	165/190	1.00	Reference		1.00	Reference	
2.64-4.76 pg/mL	176/188	1.27	(1.03-1.58)	.026	1.24	(0.96-1.61)	.100
>4.76 pg/mL	180/188	1.80	(1.46-2.23)	<.001	1.60	(1.22-2.09)	.001
Cystatin C, tertiles[a]							
<1.51 mg/dL	168/184	1.00	Reference		1.00	Reference	
1.51-1.93 mg/dL	162/181	0.95	(0.76-1.18)	.632	1.15	(0.88-1.51)	.308
>1.93 mg/dL	162/176	1.24	(0.99-1.54)	.052	1.64	(1.12-2.39)	.010
Cholinesterase, tertiles[a]							
<172 U/L	193/203	1.00	Reference		1.00	Reference	
172-210 U/L	174/195	0.60	(0.49-0.74)	<.001	0.79	(0.61-1.03)	.081
>210 U/L	169/194	0.53	(0.43-0.65)	<.001	0.68	(0.51-0.90)	.007

NT-proBNP, N-terminal pro-brain natriuretic peptide.

Age-group specific hazard ratios (HR) and 95% confidence intervals (CIs), and two-sided P values were calculated with the use of multivariate Cox proportional hazard models adjusted for age, sex, educational status, current smoking, history of cardiovascular disease, hypertension, hyperlipidemia, diabetes mellitus, chronic kidney disease (stage 3b-5), elevated CRP (≥0.3 mg/dL), major ECG abnormality, cardiovascular medication and low plasma albumin (<3.5 g/dL).

[a] Biomarkers were entered into the multivariate models as age group-specific tertiles.

[b] NT-proBNP was entered into the multivariate models as dichotomized variables with a conventional cutoff point of heart failure in the very old (≥1800 pg/mL)

[c] CRP (≥0.3 mg/dL) was excluded for associations of interleukin-6 with mortality, because it is a downstream biomarker of interleukin-6 pathway.[1]

Supplementary Table 10. Cross-sectional comparison of the levels of prognostic biomarkers and albumin at enrollment among decedent centenarian categories

Age at enrollment	Decedent Centenarians (died between 100-104 years)	Decedent Semi-supercentenarians (died between 105-109 years)	Decedent Supercentenarians (died at 110 years or older)	P
a. ln_NT-proBNP[a]				
100-104 years	6.66 ± 1.10	6.50 ± 0.93	5.38 ± 0.55	0.044
105-109 years	NA	7.05 ± 0.99	6.51 ± 1.04	<0.001
110 years or older	NA	NA	7.38 ± 0.81	NA
b. In_interleukin-6[a]				
100-104 years	1.26 ± 0.63	1.13 ± 0.54	0.83 ± 0.25	0.097
105-109 years	NA	1.40 ± 0.74	1.19 ± 0.65	0.010
110 years or older	NA	NA	1.74 ± 0.91	NA
c. Cystatin C, mg/dL				
100-104 years	1.64 ± 0.53	1.62 ± 0.52	1.27 ± 0.08	0.305
105-109 years	NA	1.82 ± 0.56	1.72 ± 0.50	0.133
110 years or older	NA	NA	1.87 ± 0.61	NA
d. Cholinesterase, IU/L				
100-104 years	211 ± 58	216 ± 52	258 ± 37	0.178
105-109 years	NA	190 ± 60	210 ± 55	0.002
110 years or older	NA	NA	178 ± 44	NA
e. Albumin, g/dL				
100-104 years	3.60 ± 0.43	3.68 ± 0.37	3.92 ± 0.44	0.095
105-109 years	NA	3.33 ± 0.46	3.48 ± 0.37	0.007
110 years or older	NA	NA	3.21 ± 0.39	NA

NA, not applicable; *NT-proBNP,* N-terminal pro-brain natriuretic peptide.
Plus-minus values are means ± SD. All the biomarkers were assessed at the time of enrollment. Differences between the decedent centenarian categories are calculated by one-way ANOVA (among three groups) or Mann-Whitney U test (between two groups).
[a] Logarithmic transformation was performed on a natural log scale for NT-proBNP and interleukin-6.

Supplementary References

1. Ridker PM. From C-Reactive Protein to Interleukin-6 to Interleukin-1: Moving Upstream To Identify Novel Targets for Atheroprotection. Circ Res;118(1):145-56. doi: 10.1161/CIRCRESAHA.115.306656.
2. Arai, Y. et al. Inflammation, but not telomere length, predicts successful ageing at extreme old age: a longitudinal study of semi-supercentenarians. EBioMedicine 2, 1549-1558 (2015).
