# Supplementary material for: Associations of cardiovascular biomarkers and plasma albumin with exceptional survival to the highest ages
Source: Nat Commun. 2020 Jul 30;11:3820. doi: 10.1038/s41467-020-17636-0 (PMC7393489; doi:10.1038/s41467-020-17636-0)
Supplement: Supplementary file 3 — Reporting Summary [file 41467_2020_17636_MOESM3_ESM.pdf]

nature research

Corresponding author(s): Yasumichi Arai

Last updated by author(s): May 27, 2020

Reporting Summary

Nature Research wishes to improve the reproducibility of the work that we publish. This form provides structure for consistency and transparency in reporting. For further information on Nature Research policies, see our Editorial Policies and the Editorial Policy Checklist.

Statistics

For all statistical analyses, confirm that the following items are present in the figure legend, table legend, main text, or Methods section.

n/a	Confirmed	
☐	☒	The exact sample size (*n*) for each experimental group/condition, given as a discrete number and unit of measurement
☐	☒	A statement on whether measurements were taken from distinct samples or whether the same sample was measured repeatedly
☐	☒	The statistical test(s) used AND whether they are one- or two-sided<br>*Only common tests should be described solely by name; describe more complex techniques in the Methods section.*
☐	☒	A description of all covariates tested
☐	☒	A description of any assumptions or corrections, such as tests of normality and adjustment for multiple comparisons
☐	☒	A full description of the statistical parameters including central tendency (e.g. means) or other basic estimates (e.g. regression coefficient) AND variation (e.g. standard deviation) or associated estimates of uncertainty (e.g. confidence intervals)
☐	☒	For null hypothesis testing, the test statistic (e.g. *F*, *t*, *r*) with confidence intervals, effect sizes, degrees of freedom and *P* value noted<br>*Give P values as exact values whenever suitable.*
☒	☐	For Bayesian analysis, information on the choice of priors and Markov chain Monte Carlo settings
☐	☒	For hierarchical and complex designs, identification of the appropriate level for tests and full reporting of outcomes
☐	☒	Estimates of effect sizes (e.g. Cohen's *d*, Pearson's *r*), indicating how they were calculated

Our web collection on statistics for biologists contains articles on many of the points above.

Software and code

Policy information about availability of computer code

Data collection	No software was used to collect the data.
Data analysis	Analyses were performed using STATA SE 13 software (Stata Corp LP, College Station, TX, USA), and R (version 3.4.3. and glmnet package for LASSO selection; version 3.6.1. and survcomp package (version 1.36.1) for calculating C-index and optimisms). All P values were two-tailed except for the C-index based on Cox hazard models, and a P value <0.05 was considered statistically significant.

For manuscripts utilizing custom algorithms or software that are central to the research but not yet described in published literature, software must be made available to editors and reviewers. We strongly encourage code deposition in a community repository (e.g. GitHub). See the Nature Research guidelines for submitting code & software for further information.

Data

Policy information about availability of data

All manuscripts must include a data availability statement. This statement should provide the following information, where applicable:

- Accession codes, unique identifiers, or web links for publicly available datasets
- A list of figures that have associated raw data
- A description of any restrictions on data availability

The datasets analyzed in the current study have ethical and legal restrictions for public deposition due to the inclusion of sensitive information from a vulnerable population. The data will be available upon request with an appropriate research arrangement with approval of the Research Ethics Committee of Keio University School of Medicine for Clinical Research. Thus, to request the data, please contact Dr. Yasumichi Arai (corresponding author) via e-mail: yasumich@keio.jp.

Field-specific reporting

Please select the one below that is the best fit for your research. If you are not sure, read the appropriate sections before making your selection.

- [x] Life sciences
- [ ] Behavioural & social sciences
- [ ] Ecological, evolutionary & environmental sciences

For a reference copy of the document with all sections, see nature.com/documents/nr-reporting-summary-flat.pdf

Life sciences study design

All studies must disclose on these points even when the disclosure is negative.

Sample size	A total of 1,427 oldest-old individuals, including 36 supercentenarians (aged ≥ 110 years at enrollment), 572 semi-supercentenarians (105–109 years), 288 centenarians (100–104 years), and 531 very old persons (85–99 years) were enrolled in the present study. Because of the extremely low prevalence of the oldest individuals (e.g., prevalence of individuals >105 years was 2.0 in 100 000 population in 2010), we reached maximum sample size during the sampling period. We enrolled more individuals >105 years (N=608) than any previous study.
Data exclusions	Some data relating to each circulating biomarker are inevitably missing, due to variation in the biobanking of samples. We excluded parameters with missing variables from our mortality analysis, rather than imputing them. The numbers of valid measurements are presented in all Tables and Figures, including supplemental materials.
Replication	Internal evaluation of results robustness was assessed using sensitivity analyses or bootstrap analyses with 100,000 resampling, whenever applicable. External replication of our results could not be performed because there is no equivalent dataset of the oldest population (i.e. supercentenarians).
Randomization	Because this is a longitudinal observation study, randomization was not feasible. Multivariate analyses were controlled for potential confounders of all-cause mortality at advanced age, including sex, age, educational status, and current smoking, traditional cardiovascular risk factors (history of cardiovascular disease, hypertension, hyperlipidemia, diabetes mellitus, chronic kidney disease [stage 3b-5]major ECG abnormality, and cardiovascular medication, CRP >0.3mg/dL), and plasma albumin levels<3.5g/dL.
Blinding	Because of the observational nature of this study, blinding of research participants was not indicated. All participants were followed-up for all-cause mortality annually via telephone contact or a mail survey by our research staff, who are independent of investigators.

Reporting for specific materials, systems and methods

We require information from authors about some types of materials, experimental systems and methods used in many studies. Here, indicate whether each material, system or method listed is relevant to your study. If you are not sure if a list item applies to your research, read the appropriate section before selecting a response.

Materials & experimental systems

n/a	Involved in the study	
x		Antibodies
x		Eukaryotic cell lines
x		Palaeontology and archaeology
x		Animals and other organisms
	x	Human research participants
x		Clinical data
x		Dual use research of concern

Methods

n/a	Involved in the study	
x		ChIP-seq
x		Flow cytometry
x		MRI-based neuroimaging

Human research participants

Policy information about studies involving human research participants

Population characteristics	Supercentenarians (those aged ≥110 years) are approaching the current limit of human longevity by avoiding or surviving major illness. Identifying specific biomarkers conducive to exceptional survival may provide molecular insights into counter-regulatory mechanisms against aging-related disease. For this purpose, the analytic cohort comprised 1,427 oldest-old individuals from three longitudinal cohort studies, including 36 supercentenarians (aged ≥ 110 years at enrollment), 572 semi-supercentenarians (105–109 years), 288 centenarians (100–104 years), and 531 very old persons (85–99 years).
Recruitment	This study used data from three prospective cohort studies of the oldest old in Japan: the Tokyo Centenarian Study (TCS), Japanese Semi-supercentenarian Study (JSS), and Tokyo Oldest Old Survey on Total Health (TOOTH). The detailed recruitment scheme for each cohort was described in the main text. Given an extremely old age of our sample, there may be a healthy volunteer bias (i.e. healthier people are more likely to participate). However, we expect this effect to be similar across all the age groups studied so that they do not affect the comparison between centenarian age groups.
Ethics oversight	We have complied with all relevant regulations for work with human subjects. Written informed consent to participate in the present study was obtained either from the participants or their proxy when the individual lacked the capacity to consent. All cohort studies were approved by the ethics committee of the Keio University School of Medicine (ID: 20021020, 20022020, 20070047), and are registered in the University Hospital Medical Information Network Clinical Trial Registry as observational studies (ID: UMIN000040446, UMIN000040447, UMIN000001842).

Note that full information on the approval of the study protocol must also be provided in the manuscript.
